# Oral manifestations of undiscovered systemic amyloidosis: a series of seven cases

**DOI:** 10.11604/pamj.2024.49.89.20032

**Published:** 2024-11-24

**Authors:** Raouaa Belkacem Chebil, Mellouli Nour, Moussaoui Eya, Mrabet Sanda, Sriha Badreddine, Oualha Lamia, Douki Nabiha

**Affiliations:** 1 Department of Oral Medicine and Oral Surgery, Faculty of Dental Medicine, Monastir University, Monastir, Tunisia,; 2 LR12ES11: Laboratory of Research on Oral Health and Oro-Facial Rehabilitation, Monastir, Tunisia,; 3 Department of Nephrology, Faculty of Medicine, Sousse University, Sousse, Tunisia,; 4 Department of Cytology and Pathological Anatomy, Faculty of Medicine, Sousse University, Sousse, Tunisia

**Keywords:** Amyloidosis, oral manifestations, histology, salivary glands

## Abstract

Amyloidosis derived from abnormal extracellular fibril deposits may contribute to multiple organ dysfunctions. The recognition of amyloidosis-associated orofacial changes may be beneficial for the early diagnosis of this systemic pathology and the underlying diseases. This retrospective study aimed to determine the characteristics of orofacial amyloidosis to aid dentists in the recognition of this disease. Seven patients consulting the Department of Oral Medicine and Oral Surgery at Sahloul Hospital from January 2010 to January 2019 and diagnosed with systemic amyloidosis were included. The median age of onset, the most commonly affected sites, the predominant oral features of amyloidosis, and its complications were presented. The results of minor salivary gland and tongue biopsies were evaluated. Macroglossia was the most frequent oral lesion and it was associated with AL amyloidosis in 5 cases. Minor salivary gland biopsy was positive in four cases. We concluded that the dentist, especially the oral pathologist, has an important role in the evaluation of the local alterations that may reflect the patients´ systemic deterioration.

## Introduction

Amyloidosis is a disease characterized by systemic or localized deposition of amyloid fibrils in the extracellular spaces of organs and tissues. These amyloid deposits are identified based on their apple-green birefringence under a polarized light microscope after being stained with Congo red. Different classifications have been proposed, although the Nomenclature Committee of the International Society of Amyloidosis continues to recommend a classification system based on the chemical identity of amyloid fibril-forming proteins. Over 25 different proteins have been associated with amyloidosis [1]. There are four major categories of amyloidosis: primary systemic amyloidosis, secondary systemic amyloidosis, hereditary systemic amyloidosis, and localized amyloidosis.

The two most common forms of systemic amyloidosis are light-chain (AL) amyloidosis, with an incidence of approximately one case per 100,000 persons yearly in Western countries, and reactive amyloidosis resulting from chronic inflammatory diseases [2]. Due to a large spectrum of clinical, histological, and biochemical aspects associated with amyloidosis, its diagnosis is often delayed or missed. Consequently, the mean survival rate of patients with the systemic forms is between 5 and 15 months [3]. The head and neck amyloidosis is rarely observed. It frequently affects patients with a light chain systemic amyloidosis, mostly those harboring plasma cell dyscrasias [4]. Ninety percent of patients with systemic amyloid develop amyloid deposits in the upper aerodigestive tract. The most frequent head and neck affected sites are the larynx, orbit, sinuses, oral cavity, salivary glands, and pharynx. The larynx is rarely associated with systemic amyloidosis. In contrast, amyloid macroglossia is usually associated with systemic AL amyloidosis. Little is known about the predilections and outcomes of oral cavity-developing amyloidosis.

The current study aimed to report the clinicopathological and immunohistochemical aspects of seven cases of oral amyloidosis and to correlate these findings with the literature.

## Methods

A retrospective clinical study was conducted at the Department of Oral Medicine and Oral Surgery in Sahloul Hospital from January 2010 to January 2019. We collected patients diagnosed with oral amyloidosis based on the clinical manifestations and the histopathological findings. To confirm the diagnosis, oral biopsies were reviewed by two pathologists, and a histological diagnosis was made as per the recent criteria proposed by the Nomenclature Committee of the International Society of Amyloidosis [1].

Congo red histochemical staining was performed previously and it was positive for apple-green birefringence under polarized light in all the cases. Moreover, an immunohistochemistry assay was carried out to investigate the nature of the amyloid deposits. Immunostaining was performed by incubating primary antibodies against the kappa light chains [Bond TRU-PA0606] and lambda light chains [Bond RTU-PA0570] revealed by Leica Bond polymer Refine detection kit DS3800. The demographic data, chief complaints, medical history, oral clinical assessment, findings of systemic diseases, and results of laboratory examinations were collected.

## Results

Seven oral amyloidosis patients were initially diagnosed in our dental unit based on orofacial abnormalities. The patients included 5 men and 2 women aged between 42 and 72 years with an average of 53 years at presentation. All the patients were referred from the nephrology unit for different reasons (oral candidiasis treatment, oral infection assessment before prescribing systemic corticotherapy or tooth decay treatment). All the included patients had a medical history. Three patients were at a late stage of chronic renal failure and were treated by hemodialysis. Acute renal failure was recently diagnosed in three patients and a nephrotic syndrome was diagnosed in patient seven. Four patients were affected by multiple myeloma and two patients had a history of carpal tunnel syndrome (Table 1).

**Table 1:** clinical data of patients with oro-facial amyloidosis

P	Age years	sex	Affected sites	Oral clinical signs	Results of biological exams	Systemic diseases	Evolution
1	42	M	Tongue, labial mucosa	Macroglossia	Hb 7.4*	C R F	Died 4 months after diagnosis
				Multiple wax-like nodules	Creatinine (642**	CTS	
				Necrotic area on the tongue	P E: monoclonal peak type Ig G Kappa	MM	
						Anemia	
2	63	F	Tongue, buccal and labial mucosa, and gingiva	Macroglossia	Hb (9.4*)	CRF	alive
				Multiple wax-like nodules	Creatinine 492**	Kidney transplant	
					P E: monoclonal peak type lambda	MM	
						Osteomalacia	
						Anemia	
3	57	F	Tongue, buccal mucosa	Macroglossia	Hb (8*)	ARF	alive
				Multiple wax-like nodules	Creatinine 1922**	MM	
					Troponin 550 ng/l	CTS	
					P E: monoclonal peak type Ig G Kappa	Anemia	
4	45	M	Tongue	Ulceration	Creatinine 742**	CRF	-
					Hb 7.2*	Hypertension	
						Hepatitis B	
5	71	M	Buccal and labial mucosa	Multiple nodules	Hb (9*)	ARF	_
					Creatinine 922**	Hypertension	
					Troponin 510 ng/l	Anemia	
					P E: monoclonal peak type Ig G Kappa	Prostate hypertrophy	
6	72	M	Tongue	Macroglossia	Creatinine 113**	Hypertension	_
				Multiple nodules	P E: monoclonal peak type Ig G Kappa	MM	
						Prostate hypertrophy	
						ARF	
7	67	M	Tongue, labial, buccal mucosa, and orbital skin	Macroglossia	Creatinine 54**	Hypothyroidism	alive
				Petechia	P E: monoclonal peak type Ig G Kappa α 2 (10.7)	ARF	
				Purple nodules and surfaces		COBP	
				Palpebral			
				Ecchymosis			

* g/dl, ** µmol/L, M: male, F: female, P E: Protein Electrophoresis, CRF: Chronic renal failure, COBP Chronic obstructive broncho-pneumopathy, ARF: Acute renal failure, MM: Multiple myeloma, CTS carpal tunnel syndrome; P: patient

**Figure 1:**
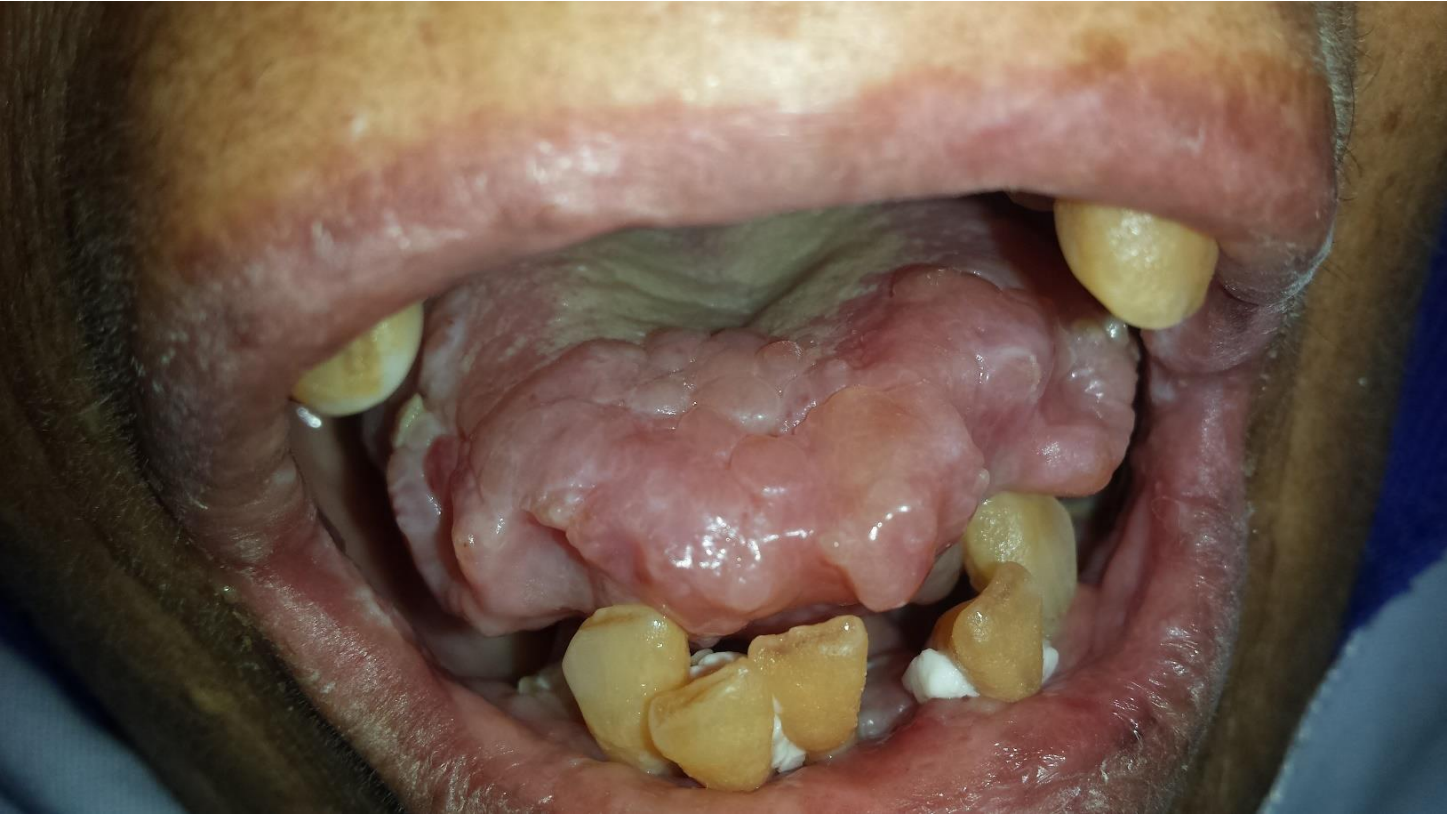
macroglossia with multiple, painless, and well-circumscribed hard nodules involving the tongue, labial, and buccal mucosa in subject 2

**Figure 2:**
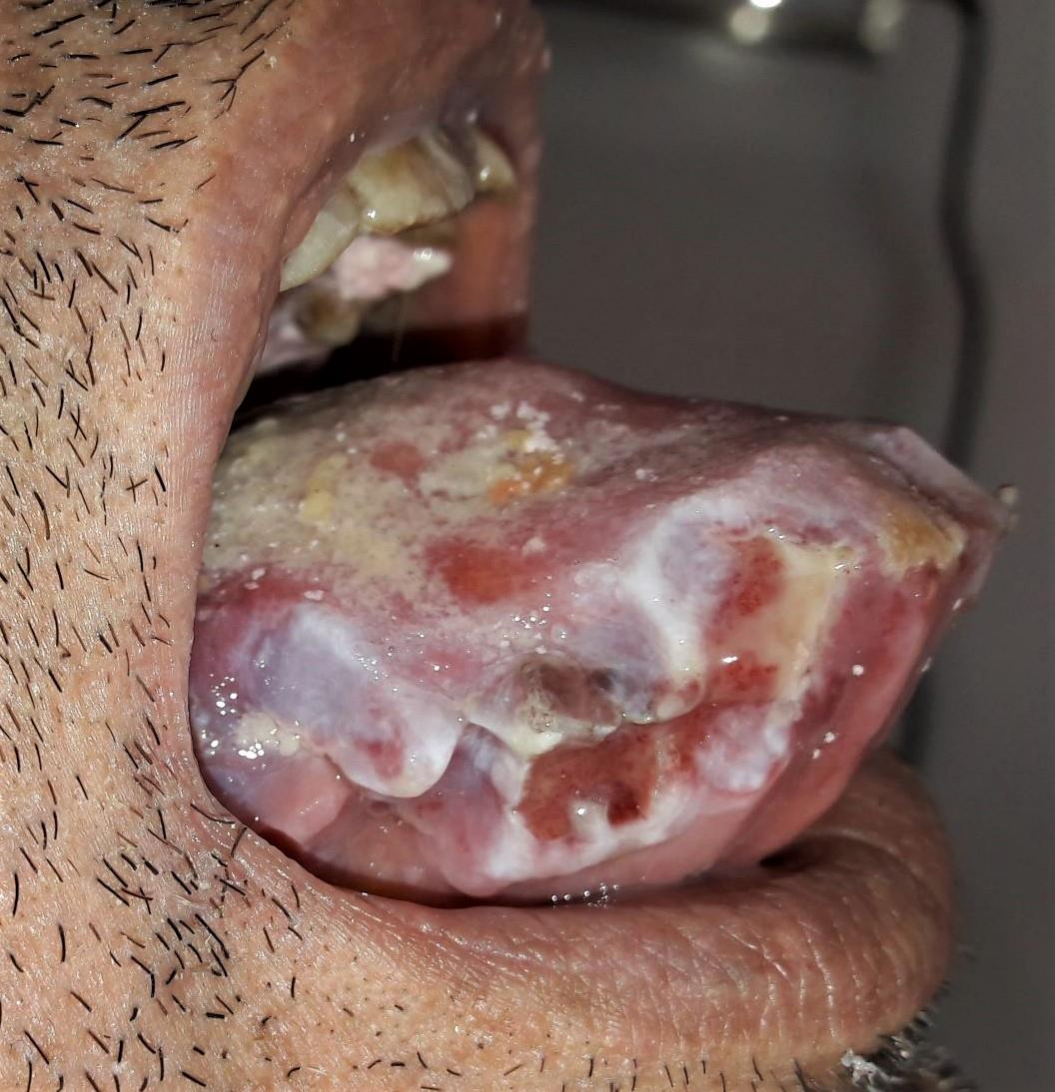
macroglossia with necrotic areas in patient one

**Figure 3:**
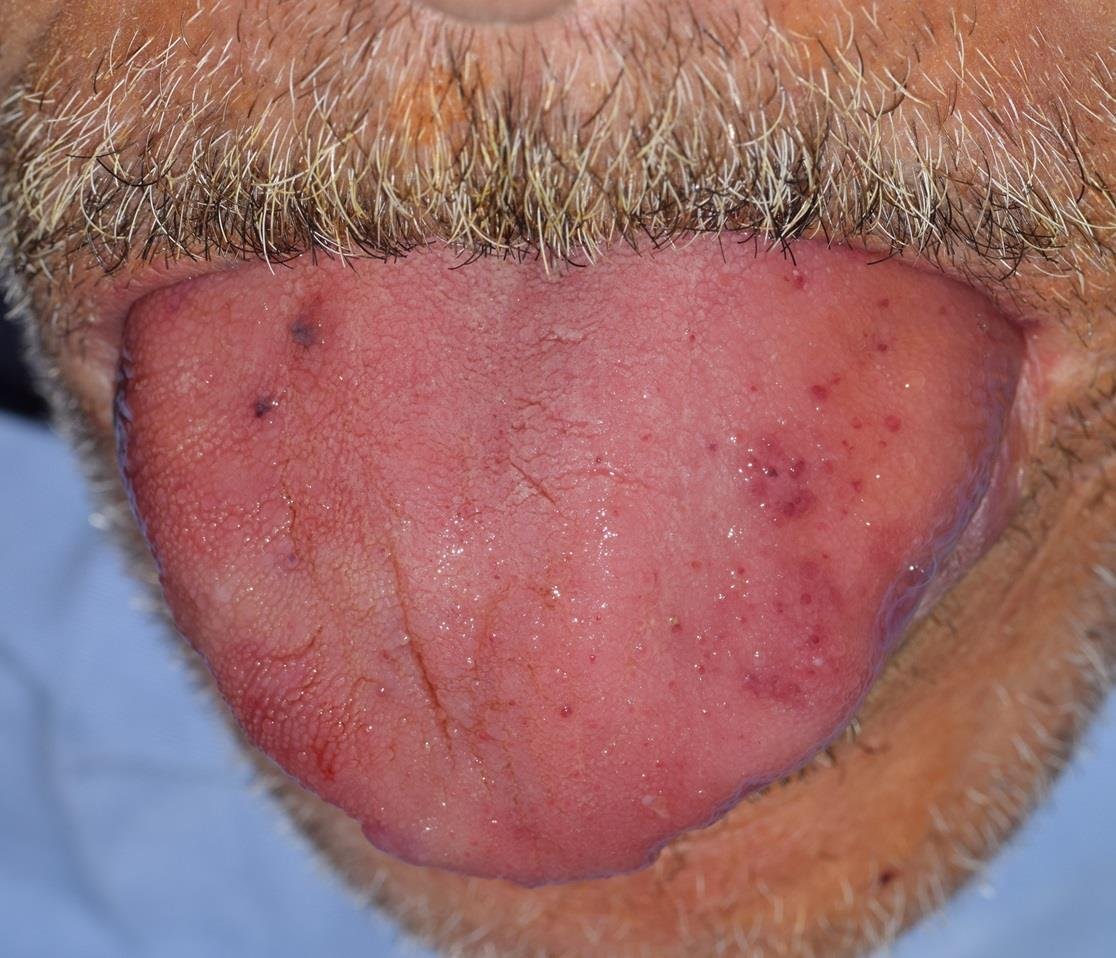
macroglossia with petechia in its dorsum in patient seven

The initial clinical signs correlated with the site of the amyloid deposits. Macroglossia revealed tongue infiltration in six cases. The second most common affected sites were the buccal and labial mucosa (4/7). Only one patient developed gingival lesions. Two patients had multiple affected sites in the orofacial region. The patients with macroglossia had firm to rubbery tongues on palpation and they typically demonstrated scalloping of the lateral border of the tongue due to indentations from the teeth (Figure 1). Tongue enlargement was associated with necrotic areas on the lateral borders in patient six (Figure 2) and it was associated with petechiae in its dorsum in patient 7 (Figure 3).

**Figure 4:**
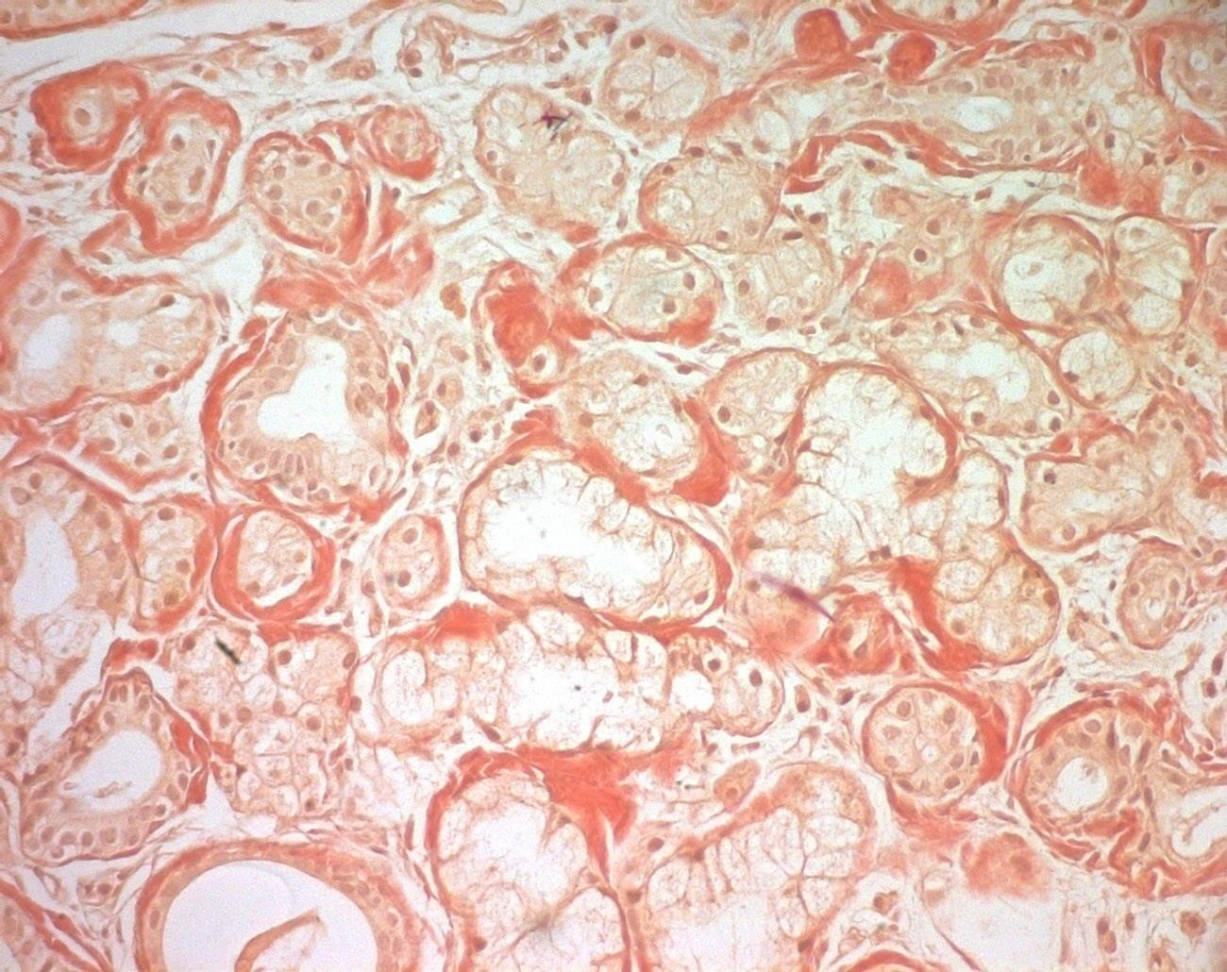
photomicrograph of the lesion showing deposition of amyloid from the minor salivary glands in case of systemic AL amyloidosis (Congo red staining, magnification 200×); (the photomicrographs were taken from the pathological slices from patient seven)

**Figure 5:**
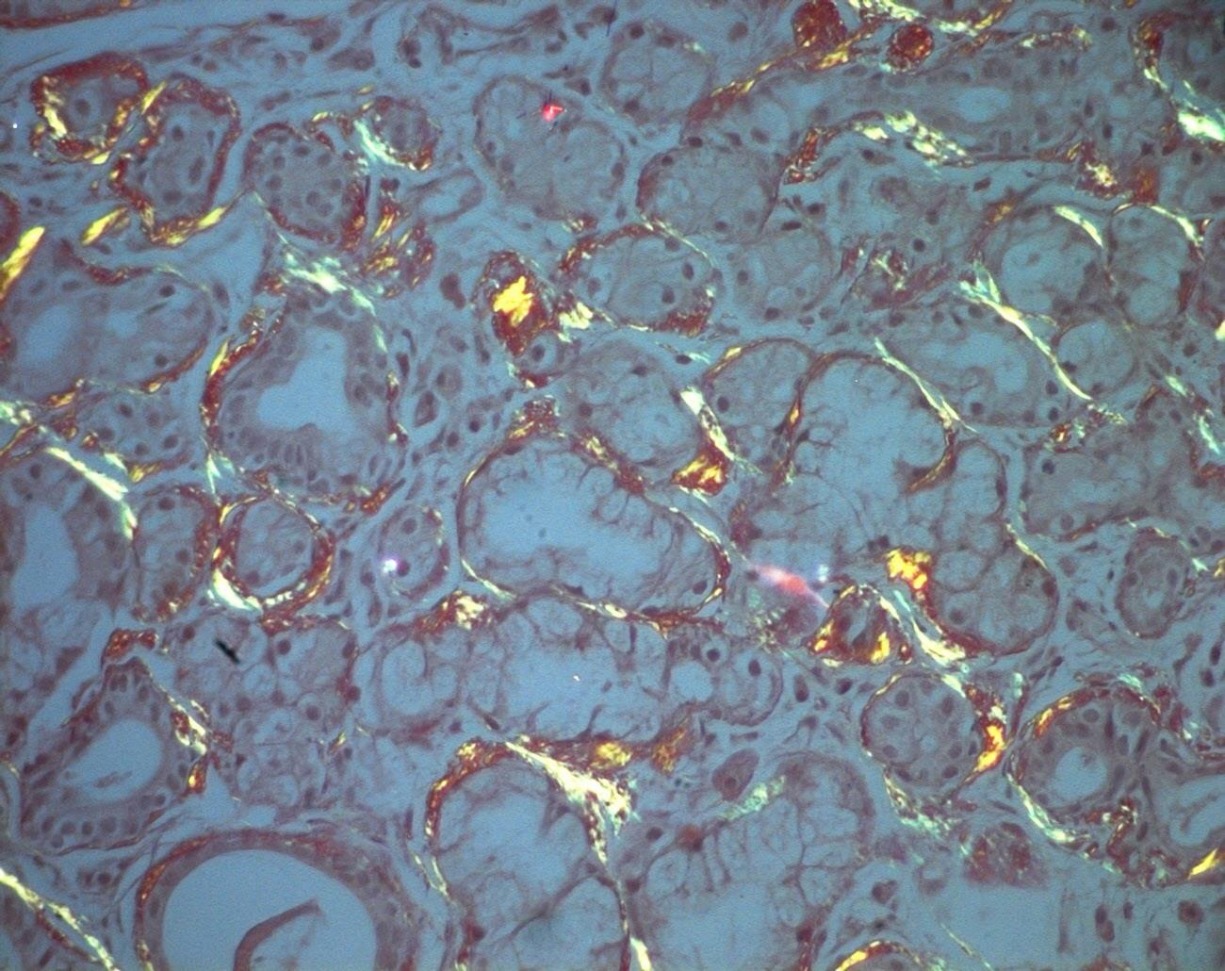
photomicrograph of the lesion showing the surface epithelium; deposits of amyloid displaying characteristic apple-green birefringence by polarized Light microscopy (Congo red stain, magnification 200×); (the photomicrographs were taken from the pathological slices from patient seven)

The buccal and labial lesions mainly presented with multiple, painless, and well-circumscribed fibrous nodules that were 3-10 mm in diameter. Ecchymosis and purple nodules that did not disappear under pressure were also present in patient seven. In this same patient, bilateral periorbital purpura was also observed. Biological examinations confirmed anemia in 5 patients and showed monoclonal peaks of light chains kappa or lambda in five patients. The troponin level was investigated in patient 3 and it showed heart involvement (cardiac insufficiency). All the tissue samples were obtained by incisional biopsy from the minor salivary glands and in some cases from the infiltrated tongue. Minor salivary gland biopsy (MSGB) was positive in four cases (57.14%) (Figure 4, Figure 5).

**Figure 6:**
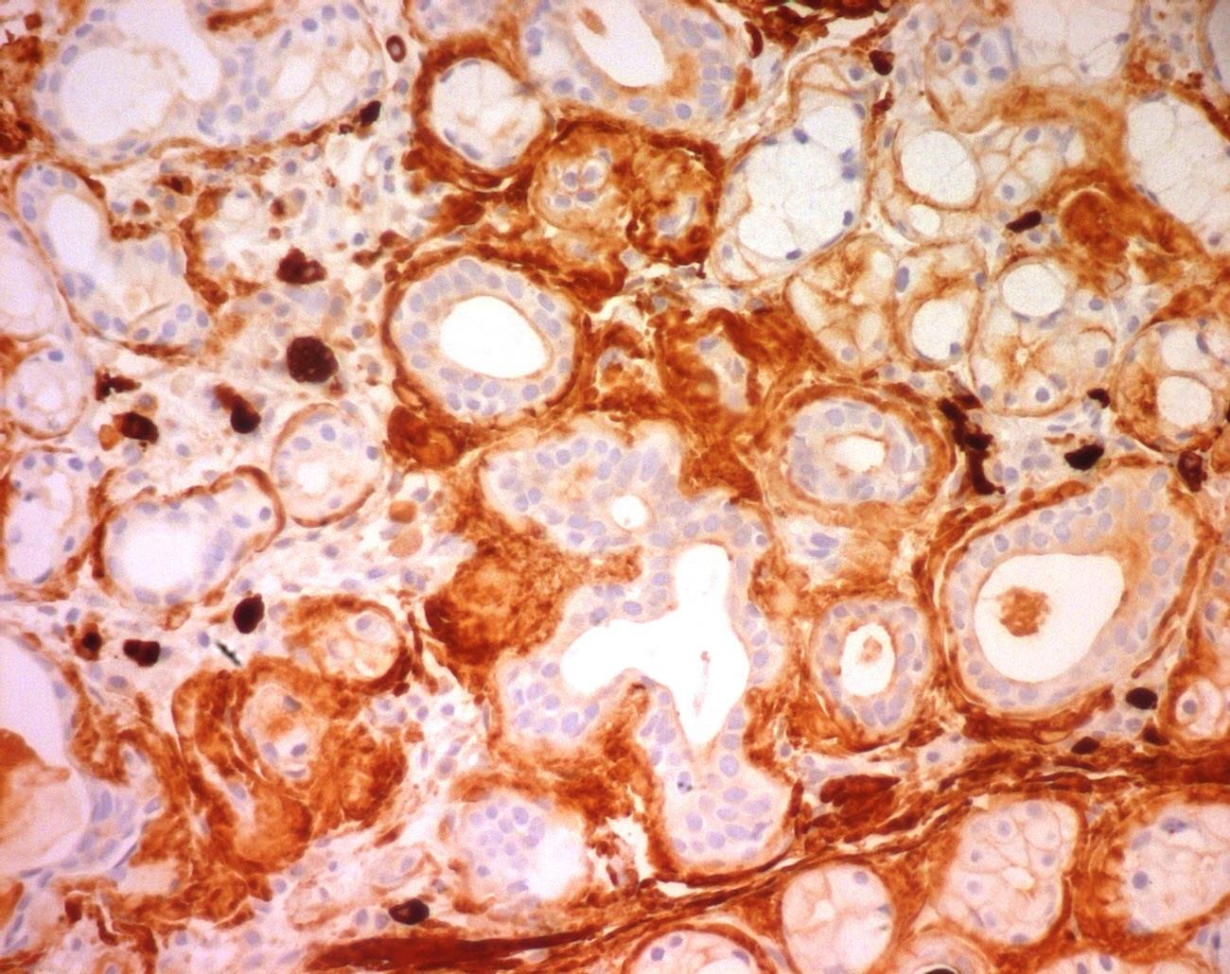
positive λ light chain immunohistochemical staining of the amyloid deposit in the minor salivary gland tissue of patient seven (magnification 200×)

**Figure 7:**
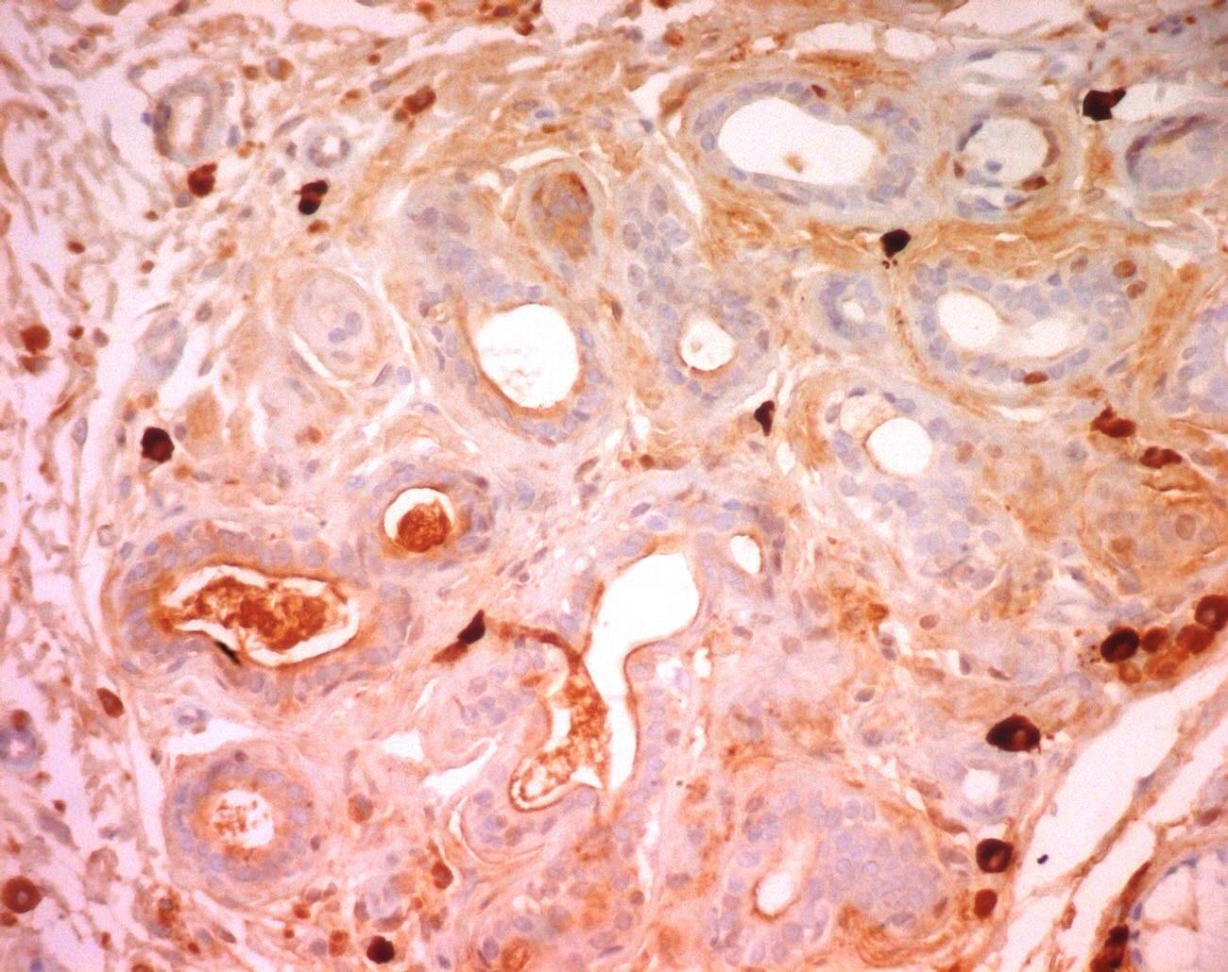
negative κ light chain immunohistochemical staining in the amyloid deposit in the minor salivary gland (magnification 200×); (the photomicrographs were taken from the pathological slices from patient seven)

A tongue biopsy was performed for 4 patients and was positive in all the cases. A kidney or liver biopsies were performed for the patients having negative MSGB and not having a tongue biopsy (Table 2). In total, all the included patients were diagnosed with systemic amyloidosis. Five patients had AL amyloidosis with positive λ light chain and negative κ light chain immunohistochemical staining in the amyloid deposit (Figure 6, Figure 7). The amyloidosis type was undetermined in two cases due to the absence of enough material to perform an immunohistochemical characterization. Two systemic amyloidosis-affected patients were deceased as a consequence of their disease. The follow-up was lost for two patients. All the patients had no radiation or major trauma to the involved sites of amyloid lesions.

**Table 2:** biopsy results and immunohistochemical characterization

Patient	Minor salivary gland biopsy	Tongue biopsy	Other biopsies	immunohistochemical characterization
1	negative	Non done	Positive kidney biopsy	AL amyloidosis
2	positive	positive	-	AL amyloidosis
3	positive	positive	Negative colon biopsy	AL amyloidosis
4	negative	positive	-	Undetermined
5	positive	Non done	-	Undetermined
6	negative	Non done	Positive kidney and liver biopsies	AL amyloidosis
7	positive	positive	-	AL amyloidosis

## Discussion

Amyloidosis is a heterogeneous group of diseases characterized by an extracellular deposition of a series of fibrillar proteins in the organs and tissues. These amyloid deposits are identified by their apple-green birefringence under a polarized light microscope after being stained with Congo red and by the presence of rigid, non-branching fibrils on electron microscopy [2]. There are four major categories of amyloidoses: three systemic types (primary, secondary, and hereditary systemic amyloidosis), and localized amyloidosis. The latter lesion is a rare subtype, involving a limited site without any systemic diseases, and having an excellent prognosis [5]. Once oral amyloidosis is confirmed, it is of critical importance to identify the subtype accurately. Deng *et al*. established a strategy for correctly discriminating between localized and systemic oral amyloidoses including clinical manifestations, assessment of the organ involvement, detailed investigation of plasma cell dyscrasia, and cooperation with techniques such as immunohistochemical staining and immunofluorescence assay. If systemic involvement is ruled out through the above investigations, localized oral amyloidosis could be confirmed. For all our patients, the same diagnostic process was performed and it confirmed the systemic involvement.

Several types of systemic amyloidoses have been identified. Nonetheless, the most frequent ones are AL amyloidosis (68%), AA (12%), TTR-related (hereditary 6.6% and acquired 3.2%), and other infrequent subtypes [6]. The current frequency of AL amyloidosis has remained stable over the last two decades. Conversely, a remarkably progressive decrease has been noticed in the proportion of patients with AA amyloidosis (from 32% to 6.8%), probably reflecting improvement in the treatment of inflammatory and infectious diseases [6]. In the present study, the most common type of systemic amyloidosis was AL. The type of amyloidosis was undetermined in two patients. In their case series study of 98 oro-facial amyloidosis-affected patients, Flores-Bozo *et al*. found that men are more likely to be affected than women without a significant gender difference and with an average onset age being at 49 years [7]. These epidemiological data were similarly found in this study with an average global age of the included patients being 53 years and a 5/2 gender ratio.

Immunofixation electrophoresis is required to detect monoclonal gammopathy of uncertain significance (MGUS) with an abnormal ratio of lambda: kappa free light chains. In contrast to the light chain isotype in MGUS and myeloma, AL fibrils are four times more often lambda than kappa light chains. In our case series study, five patients had MGUS. The light chain isotype was kappa in four patients suffering from multiple myeloma [7]. The clinical manifestations of systemic amyloidosis depend on the organ involved but are rarely specific. Renal involvement is commonly occurring in approximately 70% of patients resulting in progressive renal impairment [8]. In our study, all the included patients had kidney dysfunction. A nephrotic syndrome was recently diagnosed in one patient and an acute renal failure in three patients. Amyloid deposition in the heart is the most frequent cause of mortality in amyloidosis-affected patients. It typically presents a restrictive cardiomyopathy. It occurs in about 50% of patients with AL amyloidosis, probably because of myocardial cell toxicity of the amyloidogenic light chains [9]. The high troponin levels in patients 3 and 5 were correlated with a late stage of amyloidosis. These serum cardiac biomarkers are an important investigation for risk stratification and for determining the stage of AL amyloidosis. [10].

Regarding mucocutaneous manifestations, Amyloid deposits are frequently observed in the head and neck region, but they are rarely associated with a systemic form of the disease. On the other hand, when the tongue is involved, diagnosis of a systemic amyloidosis is usually expected [11]. Flores-Bozo *et al*. reported that mucocutaneous manifestations were observed in 34 patients among 98 systemic amyloidosis-affected patients and were seen primarily in the AL amyloidosis group (50%) [7]. These authors also noted that macroglossia was exclusively present in AL amyloidosis. In the present study, the tongue was the most frequently affected site, followed by the buccal and labial mucosa, and minor salivary glands. Macroglossia and multiple painless nodules were the predominant manifestations in the oral cavity. The first patient also exhibited ulcerative and necrotic foci (figure 1. B), which were similar to those reported by Lin *et al*. This author reported an unusual case of tongue necrosis, which occurred as a first sign of systemic vascular amyloidosis [12]. Our results clearly showed the predominance of tongue-arising AL amyloidosis with confirmation of light chain deposits in five cases.

The clinical features that are virtually pathognomonic of AL amyloidosis include a combination of macroglossia and periorbital purpura but these occur in less than a third of all the cases [6]. This combination was present in patient 7 and revealed AL amyloidosis associated with multiple myeloma. When the vessels are involved, patients may present with vascular disease and bleeding. This was noticed in patient 7 which presented petechiae and ecchymosis [5]. The involvement of soft tissues, apart from carpal tunnel syndrome, is almost unique to AL amyloidosis [6]. Carpal tunnel syndrome is a common early symptom in wild-type and hereditary ATTR amyloidosis. In our case series, patients 1 and 3 had a history of carpal tunnel syndrome while being affected by systemic AL amyloidosis.

Microscopic amyloid deposits are very widespread in the systemic forms of the disease and they may infiltrate minor and major salivary glands. Consequently, there is hypertrophy of the glandular parenchyma and acini atrophy leading to hyposalivation and swelling of the affected glands [13]. None of our patients complained of xerostomia, even though minor salivary gland infiltrate was confirmed in 4 patients. The Minor Salivary Gland Biopsy (MSGB) sensitivity was 57%. MSGB is a simple and innocuous high-yield alternative to avoid other dangerous organ biopsies with a reported sensitivity of approximately 80 %. Moreover, it helps in the early detection of amyloid deposition [14]. In a retrospective study, Suzuki *et al*. evaluated the usefulness of MSGB for diagnosing immunoglobulin light chain amyloidosis, by comparing bone marrow and skin biopsies in the same patient population. They found that the sensitivity for detecting amyloid deposition is the highest in biopsies of MSG at 89 %. Therefore, they recommended multiple biopsies of superficial tissues, including Minor Salivary Gland (MSG), bone marrow, and skin to increase the sensitivity for diagnosing AL amyloidosis [15].

In our study, all the biopsies taken from the infiltrated tongue were positive (4 cases). Zhang *et al*. evaluated the sensitivity of various biopsy methods for the diagnosis of systemic amyloidosis in 194 affected patients. They found that the highest sensitivity was achieved by a biopsy of the affected organs (renal, heart, and liver biopsies). Among the non-invasive biopsy methods, tongue biopsy was found to be 75% sensitive, followed by gingiva biopsy, abdominal fat pad aspiration, rectum biopsy, and bone marrow examination. A combination of tongue and abdominal fat pad biopsy yielded a detection rate of 93.1%. They concluded that a combination of multiple non-invasive biopsy methods may have a sensitivity comparable to organ biopsy and is safer and more convenient [16].

## Conclusion

Amyloidosis of the oral cavity predominantly involves the tongue, mainly manifesting as macroglossia. Amyloidosis of the tongue is associated with an occult underlying plasma cell dyscrasia, in particular myeloma. This study highlights the role of dentists in the recognition of amyloidosis-associated oral lesions that may reveal this systemic pathology and the underlying diseases during routine oral clinical examinations. Concerning oral involvement, surgical excision should be avoided as oral lesions normally have a high recurrence rate. The association of MSGB with tongue biopsy has a better sensitivity in the detection of systemic amyloidosis. Some limitations were found in this study. They were related to its retrospective nature, the small sample size and the high frequencies of nonspecific complications in older populations. Thus, generalization of the results should be made with caution.

### What is known about this topic

- It is well known that systemic amyloidosis may be revealed by oro-facial lesions;
- Macroglossia is the most common oral manifestation and is frequently associated with AL amyloidosis;
- Minor salivary gland biopsy has shown high sensitivity in detecting amyloid deposition.

### What this study adds

- In this case series study, we reported some unusual oral presentations with ulcerations and necrotic area on the dorsum of the tongue;
- Tongue biopsy results in comparison with minor salivary gland biopsy.
